# Patient use of AI chatbots: The overlooked frontline of health care

**DOI:** 10.1177/20552076261490748

**Published:** 2026-09-11

**Authors:** Matthew Hutnyan

**Affiliations:** 1 Department of Psychiatry, 12334 The University of Texas Southwestern Medical Center, Dallas, TX, USA

**Keywords:** artificial intelligence, health, medicine, technology, health informatics

## Abstract

Development of tailored AI health care applications has become a major research focus in recent years, but relatively little research has addressed current patient use of general-purpose AI chatbots for health-related tasks. Today, many people use AI chatbots to seek information and advice, make diagnoses, and inform or even receive intervention. The appeal is clear, but the benefits and risks are less so. While we build AI tools for the future of medicine, we must also gather empirical evidence regarding use and impact of existing tools.

In April 2024, Carey Goldberg^1^ raised a monumental question for modern clinical medicine: what do we know about patients who “take AI into their own hands” to augment their health care? Nearly three years later, the answer remains the same: next to nothing. Artificial intelligence is now ubiquitous – in health care (including the promotion, maintenance, assessment, diagnosis, monitoring, and treatment of one’s health) and daily life – and anecdotal accounts of patients using general-purpose AI chatbots for a variety of health-related tasks abound. Yet, despite the potential benefits and risks of having unfettered access to a theoretically limitless pool of information shared by human-like AI agents that have limited oversight and are known to make mistakes, there has been little systematic investigation of this phenomenon.

Use of AI chatbots is on the rise. In 2026, almost half of Americans, and two-thirds of those ages 18 to 29, reported having ever used ChatGPT, more than double 2023 estimates.^2^ About one-third of U.S. adults reported having used AI chatbots to seek information or advice about their physical health in the past year,^3^ and in one study, almost 80% of participants reported willingness to use ChatGPT for self-diagnosis of health concerns.^4^ A recent study of around 1800 U.S. adults found that 35% reported using AI tools at least once a week for mental health support,^5^ and another study showed that nearly half of the 499 participants with ongoing mental health conditions surveyed used AI chatbots for psychological support in the past year.^6^ Taken together, these results suggest that use of general-purpose AI chatbots for health-related tasks is increasingly common, especially among young adults. Patients are using these technologies to seek information and advice, make diagnoses, and inform or, in the case of mental and emotional health, receive intervention.

The appeal of AI for these purposes is understandable. ChatGPT and many other AI chatbots are free to use, can be queried at any time from any place with an internet connection, and do not require human interaction. However, accessibility comes at a cost. Troublingly, while over half of adults say they are “not confident that they can tell the difference between what is true and what is false when it comes to information from AI chatbots,” almost one-third trust them to provide reliable health information.^3,7^ Consumer difficulty evaluating the accuracy of AI-produced information is not surprising given pervasive health and technological literacy disparities and concerns related to AI chatbot hallucinations, sycophancy, and overconfidence that have not been resolved. Emerging research has shown that general-purpose AI chatbots engage in numerous concerning behaviors when conveying health information and advice, such as generating and amplifying false information, misinterpreting guidelines and evidence, providing inappropriate or fabricated citations, minimizing or magnifying reported symptoms, and reinforcing problematic beliefs and actions.^8–11^

These issues are especially important for vulnerable populations, including patients with cognitive difficulties, limited health or technological literacy, difficulties accessing care, complex medical issues, acute physical or mental health concerns, and poor prognoses. Particularly interesting (and concerning) AI chatbot use cases described in the media include youth seeking emotional support,^12^ older adults looking for companionship,^13^ people displeased with the lack of time and interest they receive from human doctors,^14^ and those desperate for answers or second opinions about their puzzling symptoms.^15^

The promise of health care AI has been widely touted, and research on the development of tailored AI tools for health care contexts has become a major focus of innovative researchers, academic publishers, and funding institutions. While this research is warranted, a predominant focus on future tailored applications has failed to account for the pressing need to better understand current use of general-purpose applications and the impact that they have on clinical care, patient experiences, and health outcomes today. If we continue to pay relatively little attention to present patient-AI interactions, we may miss the emergence and rise of new technologically mediated issues related to help seeking, misinformation, and self-diagnosis and intervention. We may also overlook potential benefits, such as increased access to health information that may be tailored, improved health literacy and understanding, greater self-efficacy, and readily available psychological support. To best serve our patients, we must invest resources into understanding how they use AI chatbots and the impact they have today while continuing to develop innovative tailored applications to improve health care for all in the future.

Some of the questions that may guide future research in this area span key domains, including the following: users and patterns of use (e.g., Who is using general-purpose AI chatbots for health-related purposes? What health-related tasks are chatbots used for?); motivations and interaction processes (e.g., Why do people use chatbots for these tasks as opposed to traditional means of accomplishing them? How do people go about these AI interactions?); information quality and safety (e.g., Is the information obtained accurate? Is it as good or better than what would be provided by a human clinician?); effects on health care and outcomes (e.g., How does use of chatbots for these purposes impact doctor-patient interactions? How does it impact health outcomes?); and health equity (e.g., How do health and technological literacy and cultural factors impact use of chatbots for health-related purposes? How are chatbots impacting marginalized and underserved communities?).

Artificial intelligence is one of the most important frontiers of the 21^st^ century, and patient use of AI chatbots represents a frontline of health care that remains overlooked. While we build AI tools for the future of medicine, we must also gather empirical evidence regarding use of existing tools. Quantitative and qualitative insights gleaned through multidisciplinary investigations will inform health care activities at individual, community, and system levels, including counseling patients on use of chatbots, updating professional guidelines, bettering general-purpose AI chatbots, and developing tailored applications. It is time to prioritize patient use of AI chatbots in health care research.
